# Whole-Genome Sequences of Five Oyster-Associated Bacteria Show Potential for Crude Oil Hydrocarbon Degradation

**DOI:** 10.1128/genomeA.00802-13

**Published:** 2013-10-03

**Authors:** Ashvini Chauhan, Stefan Green, Ashish Pathak, Jesse Thomas, Raghavee Venkatramanan

**Affiliations:** Environmental Biotechnology Laboratory, School of the Environment, FSH Science Research Center, Florida A&M University, Tallahassee, Florida, USAa; Department of Environmental Health Science, University of Georgia, Athens, Georgia, USAb; DNA Services Facility, University of Illinois at Chicago, Chicago, IIinois, USAc

## Abstract

Draft genome sequences of oyster-associated *Pseudomonas stutzeri* strain MF28, *P. alcaligenes* strain OT69, *P. aeruginosa* strain WC55, *Stenotrophomonas maltophilia* strain MF89, and *Microbacterium maritypicum* strain MF109 are reported. Genome-wide surveys of these isolates suggest that the oyster microbiome, which remains largely understudied, has a strong potential to degrade crude oil.

## GENOME ANNOUNCEMENT

On 20 April 2010, the Deepwater Horizon (DWH) blowout released an estimated 780,000 m^3^ of crude oil into the northern Gulf of Mexico (GOM) over an 85-day period (1). Consequently, up to 80,000 square miles along the impacted area were closed to fishing, resulting in loss of food, jobs, and recreation. Specifically, in the northern GOM, oyster reefs and productive oyster harvesting areas were likely exposed for several weeks to oil from the DWH oil plume. Because of their filter feeding behavior, oysters are especially susceptible to pollutants present in the overlaying water (2, 3). As a corollary, oysters may concentrate oil-degrading bacteria from the overlaying waters. In fact, several studies have shown that the oyster microbiome consists of a diverse collection of bacteria that not only differ taxonomically from the surrounding seawater but also outnumber the water bacterial populations by several orders of magnitude (4–6), likely due to the oyster’s filter feeding processes.

Here we report the draft genome sequences of five bacterial strains that we recently isolated from tissues and mantle fluid of the Eastern oyster (*Crassostrea virginica*) and the surrounding environment, using standard enrichment techniques containing crude oil as the sole source of carbon and energy (unpublished data). The Gulf crude oil utilized to isolate these strains primarily consists of aliphatic C_6_–C_35_ and polycyclic aromatic hydrocarbons (PAHs), especially C_7_–C_35_ compounds (7). We surveyed genome sequences of the oyster-associated isolates (Table 1), and based on the abundance of putative genes involved in xenobiotic degradation and metabolism encoded by these isolates, it appears that the oyster reef ecosystem is well poised to degrade crude oil hydrocarbons.

**TABLE 1:** Summary of the whole-genome sequence information of five oyster-associated bacterial isolates having the ability to degrade crude oil hydrocarbons

Isolate	Isolation source^*a*^	GenBank accession no.	No. of contigs	Length of assembly (bp)	*N*_50_ (bp)	GC content (%)	Total no. of putative genes	Total no. of putative genes for xenobiotic degradation and metabolism
*P. stutzeri* MF28	Mantle fluid	ATAR00000000	91	4,943,564	128,670	62.29	4,630	813
*P. alcaligenes* OT69	Oyster tissue	ATCP00000000	223	7,029,758	96,981	65.96	6,543	976
*P. aeruginosa* WC55	Water column	ATAQ00000000	342	6,844,176	92,012	66.14	6,650	958
*S. maltophilia* MF89	Mantle fluid	ATAP00000000	209	4,649,035	69,908	66.18	4,376	619
*M. maritypicum* MF109	Mantle fluid	ATAO00000000	260	3,996,920	158,001	68.24	4,094	608

a Oysters from Apalachicola Bay, FL, were collected using a tong. The sampling site (Dry Bar, 29°40.425'N, 85°03.406'W) is located within the Apalachicola river’s hydrologic discharge channel just southwest of the river mouth (11) and is the most productive oyster harvesting area in Florida. Oysters were culled on the boat, and 20 adult oysters of approximately the same size were collected. Additionally, 1 liter of overlaying water from the oyster reef was collected in a sterile bottle. Samples were stored on ice and transported to Florida A&M University for further processing. In the laboratory, oysters were rinsed using sterile 0.85% NaCl to remove debris and shell biofilm and each oyster was carefully shucked using sterile knives. Prior to collection of the oyster tissues, mantle fluid from the oysters was collected using sterile syringes. The details of further processing of these samples to isolate the oil-degrading microorganisms will be reported in a forthcoming manuscript.

Briefly, genomic DNA extracted from each isolate was prepared for sequencing on an Illumina HiSeq2000 instrument as described previously (8). *De novo* assembly of paired-end reads was performed within the software package CLC Genomics Workbench v 6.0 (CLCbio, Cambridge, MA). Contigs were successfully used for annotation and gene prediction by IMG ER (9), RAST (10), and NCBI’s Prokaryotic Genomes Automatic Annotation Pipeline (PGAAP), version 2.0. The suite of metabolic and catabolic degradative genes present in the isolated strains were further assessed and compared using the KEGG classification provided by IMG ER. Details of the draft assemblies are presented in Table 1. These organisms are taxonomically affiliated primarily with *Gammaproteobacteria* from the genera *Pseudomonas* and *Stenotrophomonas*, but a single isolate from the actinobacterial lineage *Microbacterium* was also recovered. Genome sizes were variable, ranging from approximately 4.65 to 7.03 Mb (Table 1). The relative abundances of putative genes for xenobiotic degradation and metabolism from the five isolates were fairly constant and ranged from 14.1 to 17.6% of all identified genes. Some examples of genes found to be common between the isolated strains include those involved in the degradative pathways of toluene, xylene, and PAHs, as well as those for the degradation of chloroalkane, chloroalkene, and nitrotoluene, respectively.

### Nucleotide sequence accession numbers.

The draft genome sequences of the strains obtained in this study have been deposited as whole-genome shotgun projects in GenBank under the accession numbers ATAQ00000000 (*Pseudomonas aeruginosa* WC55), ATCP00000000 (*Pseudomonas alcaligenes* OT69), ATAR00000000 (*Pseudomonas stutzeri* MF28), ATAP00000000 (*Stenotrophomonas maltophilia* MF89), and ATAO00000000 (*Microbacterium maritypicum* MF109).
